# Target HER four in breast cancer?

**DOI:** 10.18632/oncotarget.26867

**Published:** 2019-05-07

**Authors:** Gero Brockhoff

**Affiliations:** ^1^ Department of Gynecology and Obstetrics, University Medical Center Regensburg, Regensburg, Germany

**Keywords:** HER4, breast cancer

## Abstract

The HER4 receptor tyrosine kinase is known to have promiscuous activity in malignant cells, last but not least in breast cancer. Evidently, the prognostic and predictive impact of HER4 expression depends on the expression of different receptor isotypes, the way of receptor activation (ligand dependent vs. independent), and on the complex interaction of the HER4 intracellular domain (4ICD) with intracellular regulative molecules which results in either oncogenic or rather tumor suppressive HER4 activity. Recent data suggest that HER4 unfavorably affects the endocrine treatment of postmenopausal breast cancer patients with tamoxifen and therefore might represent an additional therapeutic target in luminal breast cancer.

The Human Epidermal Growth Factor Related (HER) receptor family comprises at least four cognate receptor tyrosine kinases (RTKs, namely HER1, HER2, HER3, and HER4) which - as a functional unit - drive breast cancer disease and have prognostic and predictive impact [1]. While HER2 represents the main therapeutic target its relatives HER1, HER2, and HER3 play a subordinate role for clinical care of breast cancer disease [2]. However, there is substantial preclinical and clinical evidence that HER1 and HER3 not only have negative prognostic impact on the course and outcome of breast cancer disease but also impair the therapeutic efficiency of an anti-HER2 targeting of HER2-positive breast cancer, predominantly carried out with trastuzumab. Accordingly, a number of strategies by targeting two or more HER receptors at the same time or targeting HER2 and downstream signaling pathways have been developed to compensate for a reduced efficiency of an anti-HER2 treatment [3–5]. 

Remarkably, HER4 plays an extraordinary role in the context of HER receptor coexpression and interaction [6]. Upon its discovery and original description [7] HER4 has been found to contribute to the development and differentiation of the mammary gland by a strictly controlled spatiotemporal expression [8, 9]. However, its role in malignant tissues, in particular in breast cancer, is evidently ambivalent. In early years HER4 has been attested a favorable impact on the course of breast cancer disease [10, 11]. An advantageous impact of HER4 expression in breast cancer has been mechanistically attributed to a ligand (i.e., heregulin) dependent receptor activation and subsequent trigger of differentiation pathways that in turn antagonize oncogenic cellular features generated not only but also by other coexpressed HER receptor family members, above all HER2 [12, 13]. A HER4 mediated differentiation process is primarily triggered by the two ligands neuregulin 3 and 4 which specifically bind to HER4 but not to related receptors [14]. (The terms “neuregulin” and “heregulin” refer equally to HER receptor specific ligands).

In contrast to its differentiation enhancing activity Her4 has also been shown to promote the proliferation of breast cancer cells [15, 16]. It has been experimentally demonstrated that the molecular mode of HER4 action is determined on the one hand by an isotype specific expression (JMa/CYT-1, JM-a/CYT-2, JM-b/CYT-1, and JM-b/CYT-2, respectively) [17] and on the other hand by a ligand dependent and independent receptor activation [15, 18]. The latter activation mode is related to the activity of Tumor Necrosis Factor Alpha-Converting Enzyme (TACE) and γ-Secretase which cause the release of an HER4 intracellular domain (4ICD) that can create either pro-apoptotic or pro-proliferative activity [19–21]. The respective intracellular activity of 4ICD is in turn elicited by its interaction with, for example, a signal transducer and activator of transcription 5 (stat5), ww domain-containing oxidoreductase (wwox), yes associated protein (yap), pro- or anti-apoptotic molecules as B-cell lymphoma 2 (bcl2), bcl1-antagonist/killer 1 (bak), and other molecules that regulate cell proliferation and survival [22, 23]. The promiscuous activity of 4ICD is supposed to be involved in an either favorable or unfavorable course of disease. Overall, the prognostic impact of HER4 seems to differ in respective taxonomic breast cancer sub-entities, i.e., luminal, HER2-positive, triple negative or basal like breast cancer since it is affected by the presence (or absence) of HER2, ER, and other subtype specific regulatory molecules [23–25]. 

Notably, HER4 does not only affect tumor cell growth and survival but also appears to significantly impact antigen specific tumor treatments by definite intracellular molecule interaction [26]. In this regard the subcellular localization and activity of the 4ICD receptor domain, i.e., nuclear vs. cytoplasmic, seems to play a crucial role [15, 27, 28]. More specifically, the treatment efficiency of HER2-positive breast cancers with trastuzumab [28] and the endocrine treatment of estrogen receptor (ER) positive breast cancers with tamoxifen seems to depend to some extent of the intracellular/cytoplasmic 4ICD localization ratio, even though the available data are not completely consistent. Years ago, HER4 (i.e., 4ICD) has already been shown to co-activate estrogen receptor related transcription and thereby to promote tumor cell growth and proliferation [15, 29]. Based on this finding and other evidences we recently demonstrated an improved outcome of ER positive and tamoxifen treated postmenopausal breast cancer patients in the absence of HER4 expression [30]. In contrast, we found no impact of HER4 in patients treated with aromatase inhibitors which indicated some kind of 4ICD/ER/tamoxifen interaction. Accordingly, we demonstrated experimentally that a siRNA mediated HER4 knock-down in ER positive breast cancer cells results in an enhanced sensitivity to tamoxifen treatment. Overall, we revealed strong evidence that HER4 (or more specifically 4ICD) impedes the tamoxifen-estrogen receptor interaction and thereby attenuates the tamoxifen treatment efficiency. Accordingly, we provided a model of action of the molecular troika consisting of 4ICD/ER/tamoxifen that explains an improved response to tamoxifen treatment in the absence and an impaired response in the presence of HER4 [30].

The finding that HER4 impairs the tamoxifen treatment efficiency has clinical implications and facilitates extended treatment strategies that involve multiple tumor cell targeting. This can in case of ER/HER4 double positive BC potentially be accomplished by a dual targeting of both receptor types using for instance tamoxifen and an HER4 specific antibody with anti-tumorigenic activity [31, 32]. Alternatively, small molecules that inhibit the HER4 kinase activity such as neratinib or afatinib might be useful but also the sequestration of estradiol which has been shown to enhance HER4 receptor shedding by stimulating TACE activity [18] might be a reasonable therapeutic strategy. Potential combined treatment modalities, that need to be prospectively explored, are illustrated in Figure 1. Accompanying analyses addressing the multivalent and complex pattern of RTK/ER interaction will extend the understanding of treatment success and failure not only of endocrine therapies of breast cancer. Elucidating the molecular mechanisms underlying specific treatment modalities will build the basis for precision medicine for instance by the combined anti-ER and anti-HER4 targeting of luminal breast cancer.

**Figure 1:**
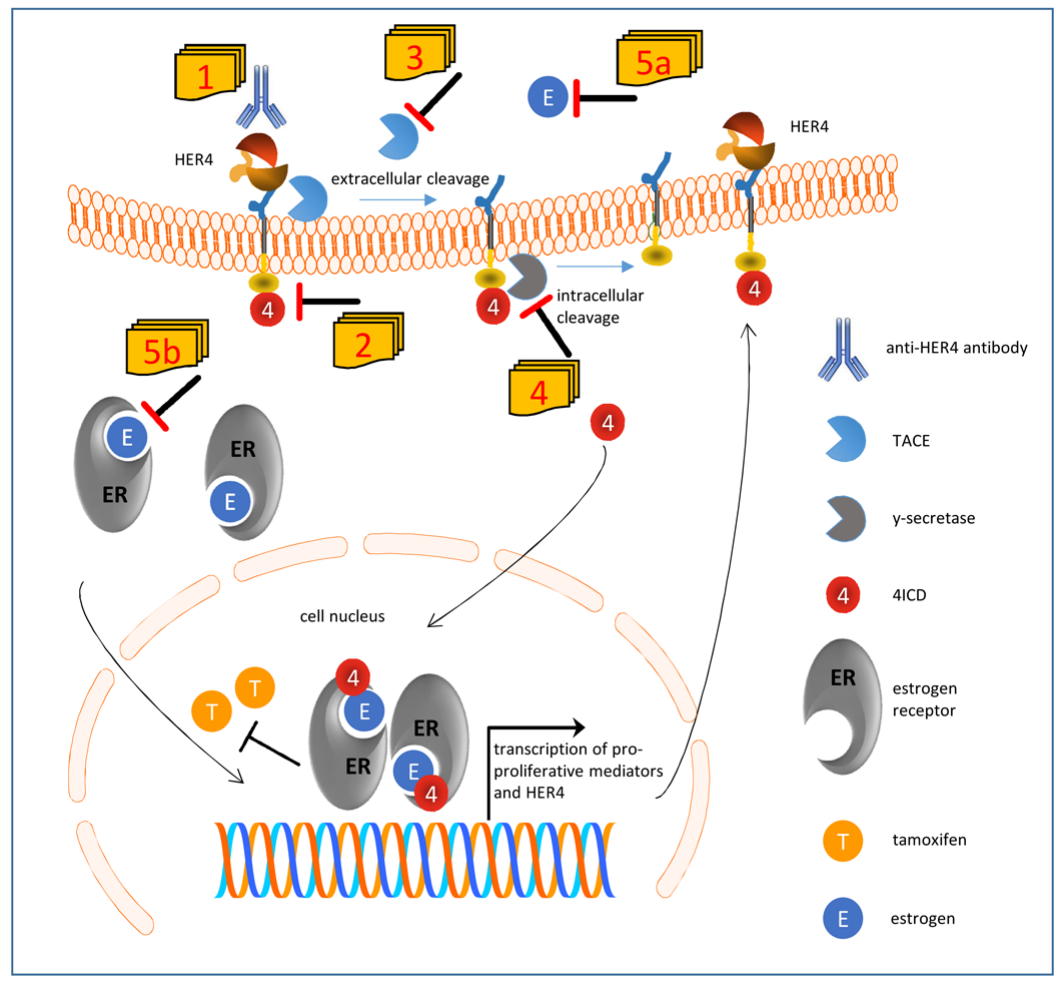
Suggested model of impaired tamoxifen treatment in the presence of HER4 receptor expression and activation and conceivable therapeutic interventions. HER4 can be processed by a two-step proteolytic activation. First tumor necrosis factor α converting enzyme (TACE) cleaves the extracellular domain, and subsequently y-secretase cleaves the intracellular domain of HER4 (4ICD) which is released into inner cell compartments. If translocated into the nucleus, 4ICD as an ER co-activator enhances the pro-proliferative effects of estrogen. Within an autoloop, 4ICD also enhances the transcription of HER4 itself. However, the 4ICD also impairs the efficient inhibition of ER by tamoxifen. The unfavorable activity of HER4 can be potentially abolished by a number of strategies: HER4 activity and cleavage can be blocked by a specific anti-HER4 antibody [1] or by an HER4 kinase inhibitor [2]. Alternatively the extracellular and intracellular cleavage can be blocked by an inhibitor of TACE [3] or γ-secretase [4], respectively. Finally, the sequestration of estradiol [5a] would also impair the ligand independent but TACE induced cleavage of HER4 and would additionally attenuate the activation of the ER [5b].
